# Patient-centered care in Israeli IVF units: divergent perceptions of patients and providers

**DOI:** 10.1186/s13584-020-00395-0

**Published:** 2020-08-06

**Authors:** Tamar R. Medina-Artom, Eli Y. Adashi

**Affiliations:** 1 Myers-JDC Brookdale Institute, PO Box 3886, 91037 Jerusalem, Israel; 2 The Bob Shapell School of Social Work, Tel Aviv University, Tel Aviv, Israel; 3 The Warren Alpert Medical School, Brown University, RI 02906 Providence, USA

**Keywords:** Patient-centered care, Fertility treatments, IVF, Patient perceptions, Provider perceptions, Emotional support, Patient wellbeing

## Abstract

**Background:**

Patient-centered care is particularly important for patients undergoing fertility treatment because of their emotional involvement and their constant contact with providers. To the best of our knowledge, to date, there have been no rigorous studies of the discrepancies between the patients’ perceptions of the care they received and the providers’ perceptions of the care that they provided, in specific dimensions and elements of patient-centered care.

**Objective:**

To compare provider and patient perceptions of the extent to which care in Israeli IVF units is patient-centered.

**Methods:**

A previously validated survey instrument was used to assess the patient and provider perceptions of ten dimensions of patient-centered care: accessibility of providers, provision of information and of explanations, communication skills of providers, patient involvement in the treatment, respect for patient values and needs, continuity and transition in treatment, professional competence, care organization, physical comfort, and emotional support. The patient survey and the provider survey were conducted in 2016–2017; both surveys were carried out in 8 of 25 hospital-based IVF units in Israel. Seventy-six providers and 524 patients (response rate 79%) participated in the surveys.

**Findings:**

The perceptions of patients and providers were similar regarding seven of the ten dimensions of patient-centered care, although there were some differences in patient vs. provider scores by unit. There were three dimensions with substantial provider-patient score differences: Moderate-sized gaps were found relative to the provision of information and explanations (1.96 vs. 2.38, on a 0–3 scale) and respect for patient values and needs (1.92 vs. 2.47). A large gap was observed relative to emotional support (0.96 vs. 2.54).

**Conclusions:**

Providers appear to underestimate the needs of fertility treatment patients for information, respect, and emotional support. The observed differences between what patients feel about their care and what providers assume they provide, especially regarding emotional support, indicates a need for ongoing, specific feedback to providers as to the patient-centeredness of the care they provide. The particularly large patient – provider gap relative to the provision of emotional support highlights the importance of increasing the attention paid to the psychological impact of fertility treatment and of giving patients an opportunity to consult a counselor who is familiar with problems associated with fertility treatments.

**Policy recommendations:**

Efforts to improve the patient-centeredness in FT should begin by establishing a national ongoing feedback mechanism, involving all 25 IVF units operating in Israel working in collaboration with the Ministry of Health. The findings from this joint effort should be shared with the public. In addition, we recommended appointing one professional in each IVF unit to be in charge of promoting improvements in the patient-centered care for that unit. Assigning a mental health professional (psychologist or social worker) to each and every IVF unit is also of crucial importance.

## Introduction

Fertility treatment (FT) necessitates constant contact for patients with providers and carries a heavy psychological and physical burden [1, 2]. We therefore believe that excellent PCC (patient-centered care) is particularly important for patients undergoing FT, similar to the importance and effectiveness of PCC for other chronic diseases [3, 4]. Fertility problems significantly reduce quality of life in patients by increasing their anxiety and depression levels [5]. Cross-sectional studies have noted that PCC in FT patients is associated with patient wellbeing, quality of life, and reduced distress [6]. In addition, women in FT prefer PCC [7], look for PCC to address the emotional burden of FT, and are willing to forego higher fertility rates to secure more PCC [8].

PCC is especially important in Israel, where the prevalence of IVF treatments is the highest in the world, and the success rate (as measured in live birth per treatment) is lower than in other advanced countries [9]. This may be related to the significant difference between Israel and other countries in the average number of treatments per woman receiving IVF care. The worldwide standard for number of treatments per woman is no more than three cycles [10], while in Israel there is no limit to the number of treatments, and women may undergo twenty or more cycles [11]. The high number of treatment cycles in Israel reflects the widespread public funding, which is the highest in the world. According to the National Health Insurance Law in Israel, all fertility treatments are covered, until the birth of a first and a second child, with an age limit of 45 for women that do not need an egg donation procedure [12].

Patient-centered care (PCC) refers to patient - provider relations that are characterized by partnership, respect and provider’s responsiveness to the patient’s preferences, needs and values [13]. There is undisputed evidence that, in a wide range of health conditions and settings, PCC is associated with patient satisfaction, well-being [14], safety, adherence to medication [15], improved clinical outcomes and reduced healthcare costs [16]. Therefore, PCC is considered to be a core element of quality of care [17] and its improvement is one of the six objectives of the IOM Health Care Quality Initiative [18]. In Israel, the Ministry of Health has included PCC as a dimension of health care quality, and it is one of the priorities of the Ministry [19].

Providers and patients might agree as to the importance of PCC in general [20], but they might also differ in their perceptions of what aspects of PCC are particularly important and in their assessments of the extent to which PCC principles are being implemented. These perceptions may be guided by their different perspectives. Indeed, in a study of patients and physicians from hospitals and health centers in Spain, discrepancies between providers and patients were found in their perspectives regarding several of the core topics included in PCC; this was most pronounced regarding the provision of information and guidance during treatment [21]. In addition, patients and providers differ in their physical and emotional experiences, expectations concerning treatment outcomes and uncertainties, time frames, and finances [22]. Studies have indicated there might be some discrepancies in PCC perceptions between patients and providers, that providers might not consider all of the dimensions of PCC as equally important [23].

Specifically with regard to FT, providers appeared to underestimate the importance of PCC for patient satisfaction [20, 24, 25], thereby suggesting that PCC in FT may be in need of improvement [26]. Specifically, the dimensions of PCC that are most important to patients might be different from those considered most important to providers (e.g. information and communication vs coordination and integration of care, respectively) [27]. Interestingly, while the data in some studies indicated that healthcare professionals underestimated their own performance [6] the data in others emphasize the opposite [25]. The aforementioned studies generally instructed the healthcare professional to answer the way their patients would have evaluated the patient-centered quality of care at their clinic.

It is not yet known what patients’ and the providers’ perceptions of PCC in Israel are, either for FT, or in general. It is also not known whether there are discrepancies in perceptions between Israeli providers and patients. More importantly, to the best of our knowledge, to date, there have been no rigorous studies of the discrepancies between the patients’ perceptions of the care they received and the providers’ perceptions of the care that they provide, in specific dimensions of patient-centered care and the even more specific elements comprising each dimension.

The purpose of the current study was to compare provider and patient perceptions of the provision of PCC in IVF units in Israel. The specific objectives were: (a) To examine patient assessment of specific dimensions of the FT experience and compare between the dimensions; (b) To examine providers assessments of those dimensions and compare between the dimensions; (c) To assess whether, and to what extent, there are gaps between FT patients and their providers in their perceptions of the provision of specific dimensions of PCC and the even more specific elements comprising each dimension.

This study was part of a broader multicenter study that was conducted using a mixed method approach, in-depth interviews with directors of IVF units and surveys of FT patients and providers, which included, in addition to PCC, other important concepts for FT patients, such as quality of life and wellbeing related to FT (forthcoming). For the purpose of the current paper we will focus on the quantitative data alone and will emphasize the differences in perceptions between FT patients and their providers.

## Methods

### Setting

The study was conducted in eight of the 25 hospital-based IVF units in Israel. We selected IVF units to reflect the diversity in their characteristics: location (center of the country or periphery according to the Peripherally Index of the Central Bureau of Statistics), magnitude (less or more than 500 treatment cycles conducted each year), and ownership (private or public hospitals). The 25 hospital-based IVF units were sorted into the five types of units that exist in Israel, where the definition of these types emerged from combinations of these characteristics. For each type, where there was a choice to be made among several units, we picked the study units randomly. Within the public hospitals, we made sure to include, government, health plan and independent hospitals. Among the for-profit hospitals, the choice of study unit was simple, as only one of the four IVF unit managers agreed to participate in the study. It is worth mentioning that the units in the public and the for-profit hospitals differ in some relevant characteristics, such as the prolonged relationship that the patients in the for-profit hospitals have with their own physicians, starting with visits in their office, followed with the procedures conducted in the hospital, vs. the relationships that the patients in the public hospitals have with the unit’s team. Having said that, it is important to note that patients in public hospitals will often be treated by the same physician, due to the small number of physicians working in the unit.

### Ethics approval and consent to participate

Data were collected after obtaining required ethical approvals. The study received approval from the Ethics Committee of both The Myers-JDC-Brookdale Institute and Tel-Aviv University. Furthermore, since the research was carried out in IVF units located in hospitals, after obtaining the consent of all IVF unit managers, approvals were received from the Ethics Committee of each of the eight hospitals involved. FT Patients received an explanation of the research and signed an informed consent form.

### Study population and study sample

The patient study population consisted of all patients who visited any of the participating IVF units during the one-year data collection period, which began in October 2016. Since a random sampling method from patient lists was not possible, due to the reluctance of the managers of the participating IVF units to reveal the list of patients who receive treatment in their unit. Therefore, a two-stage sampling method was used: unit selection, as was described above, and quota sampling in each unit, during a random day visits, up to a minimum of 50 participants in each unit. The number of questionnaires collected varied across the units, due to the long length of time required for filling out the questionnaire and the desire to give every patient the opportunity to complete the questionnaire. Therefore, we distributed questionnaires to all women present in the units on the days chosen for the study, counting the completed questionnaires only at the end of the day, while making sure the minimum quota was met.

Patients were recruited at the participating IVF units. The research team visited each unit on an average of six times until the minimum quota per unit was recruited. The provider study population consisted of all physicians, nurses and other professionals working in those units during the study period. Providers responded to the questionnaire during visits of the research team in the IVF unit, mostly during staff meetings, or through emails, with a minimum chance of hearing or seeing the other providers’ responses.

The study sample consisted of 524 FT patients and 76 providers who responded to the questionnaires. The providers sample included 22 physicians, 20 nurses, 18 administrators, 11 laboratory workers, 2 technicians, 2 auxiliary staff and a social worker. The inclusion criteria for the patient survey was being a non-pregnant woman undergoing FT in one of the participating IVF unit. We did not include spouses to avoid a possible bias from marital differences, and pregnant women to avoid a possible bias from a successful treatment. Inclusion of IVF treatments only (while excluding other fertility treatments such as ovarian stimulation or intrauterine insemination) was settled on to minimize as much as possible differences between the various types of treatments.

The patients’ survey achieved high response rate of 79%, which ranged between 71% and 86%, in the different units. See Table 1 for a detailed response rate for each IVF unit. This was calculated given the number of women who visited the participating units during the study period *and* who were approached by the study team (an effort was made to approach all visiting women). The main reasons for patient refusal to participate in the study were: lack of time, emotional state, language problems, spouse’s refusal, and fear of exposure.

**Table 1:** Valid responses to questionnaires obtained from 76 providers^a^ and 524 FT patients in eight hospital-based IVF units in Israel

IVF unit	Providers^a^	FT Patients	
	n	n	Response rate (%)
1	15	53	71
2	6	56	76
3	14	67	76
4	7	68	79
5	9	62	82
6	9	54	86
7	6	51	85
8	10	113	82
Total	76	524	79

^a^The 76 providers included: 22 physicians, 20 nurses, 18 administrators, 11 laboratory workers, 2 technicians, 2 auxiliary staff and a social worker

Table 2 specifies the demographic characteristics of the FT patients participating in the study. The patients’ median age was 35, most of them were Jews, with high education, married or living with a spouse. A considerable proportion were secular and had children at the time of the study. About a third were diagnosed with unexplained fertility problem, while about a quarter with a male or female factor causing the infertility problem.

**Table 2:** Demographic characteristics of FT patients participating in the study

Demographic characteristics of the 524 participating FT patients	
Median age (years, rang)	35 (19–50)
Religion (%)	
Jews/non-Jews	82 / 18
Level of Education (%)	
Low-Medium/High	34 / 66
Religiousness^a^ (%)	
Secular/Traditional/Religious and ultra-Orthodox	42 / 30 / 28
Marital Status (%)	
Married or living with a spouse/Divorced or single	88/12
Parenting for children (%)	
Mothers/childless women	45/55
Diagnosis given by physicians (%)	
unexplained/Male factor/female factor/both	35/26/28/11

^a^In Israel, all Jewish religious definitions refer to Orthodox Judaism. The Religious and ultra-Orthodox category presented here includes both Jewish participants and Muslim participants that defined themselves as religious. Christian participants defined themselves as either secular or traditional

### Instrument

We used a Hebrew version of the Patient-Centeredness Questionnaire (PCQ) – Infertility that was developed in the Netherlands by van Empel and her colleagues [28], combined with two additional PCC dimensions from a questionnaire developed also in the Netherlands by Dancet and her colleagues [29], The (PCQ) – Infertility is valid and reliable tool (Alpha 0.92), that has been used in other countries, e.g. in Portugal [30] and New Zealand [31]. A panel of members in the Israeli Fertility Association confirmed that the questionnaire was relevant for Israeli FT patients. The questionnaire was reviewed by FT patients in a preliminary study and was translated into Arabic and Russian as well.

The study tool included 52 questions about 10 various dimensions of the FT experience: accessibility of providers; provision of information and explanations; communication skills of providers; involvement of patient in treatment; respect of the patient’s values and needs; continuity and transition in treatment; professional competence; care organization; physical comfort; and emotional support. Table 3 displays examples of specific elements that comprise each of the 10 PCC dimensions. For example, the provision of information and explanations dimension was comprised, among other elements, of: patients receiving written information and patients receiving a scheduled overview of treatment plan.

**Table 3:** The 10 PCC dimensions and the specific elements that comprise them^a^

Patient-centered care dimensions	Examples of specific elements included in each dimension
Accessibility of providers	-Telephonic access of the hospital -Accessibility of providers for questions
Information and explanation	-Receiving written information -Receiving a scheduled overview of treatment plan
Communication skills of providers	-Honesty and clarity on what to expect of the treatments -Providers talking about patients instead of talking to them
Involvement of patient in treatment	-Openness to patient’s opinion and ideas about treatment -Opportunity to ask questions
Respect for values and needs	-Access to patients’ own medical record -Empathy with patients’ emotions and current situation
Continuity and transition in treatment	-Having a lead physician for evaluation and decision-making -Contradictory policy adhered by different providers
Professional competence	-Providers using difficult words without explaining them -Physician being well prepared for appointments
Care organization	-Waiting time between first visit and receiving treatment plan -Waiting time between two treatments
Physical comfort	-Waiting room being comfortable -Waiting time in consultation waiting room being acceptable
Emotional support^b^	-Being informed about the psychological impact of treatment -Given the opportunity to consult a counselor who was familiar with problems connected with treatment -Receiving information on support group for FT patients -Partner and or family members provided with an information brochure

^a^Van Empel and colleagues [28] and Dancet and colleagues [29]

^b^The Emotional support dimension specifies here all four elements included in this dimension

FT patients were asked to evaluate on a 0–3 scale their experience on each of the 10 dimensions. Providers were asked, on a corresponding questionnaire adapted to providers, to score on the same scale the degree of their agreement with various descriptions of the treatment given in their unit.

### Variables

The dependent variables were: providers’ and patients’ scores in the various dimensions of PCC and the gaps (in absolute values) between those scores. No gap (calculation resulting in zero) indicated an agreement between providers and FT patients in regard to the extent to which the dimension of interest characterized care in the IVF unit. A gap meant that there was a difference between the patients’ and providers’ perception of the provision of the dimension of interest, regardless to the question of who’s score was higher.

### Analysis

The patient survey and the provider survey data were analyzed quantitatively using IBM SPSS Statistics Version 24. The analysis included the calculation of scores given by FT patients and by the providers in the various dimensions of PCC, and the gaps between average FT patient scores and average provider scores. The gaps were calculated in absolute value. We then calculated these scores and gaps per participating unit. The data were weighted to reflect the differences in the number of treatment cycles conducted annually at each unit.

## Findings

**Fig. 1:**
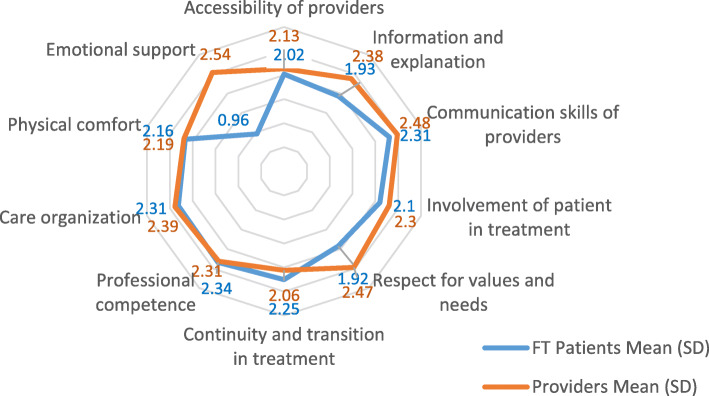
Scores^1^ on a 0–3 scale provided by FT patients and providers on their perception of the provision of the various dimensions of PCC. ^1^All scores represent significant differences at the level of *p* < 0.001, except for the scores in the Accessibility of providers and Organization of treatment dimensions that represent significant differences at the level of *p* < 0.01

FT patients indicated they felt that not all dimensions of PCC were implemented to the same extent, as can be reflected by the different scores that they gave to the 10 various PCC dimensions (See Fig. 1). FT patients gave the highest scores to three dimensions: communication skills of providers and care organization (both had a mean score of 2.31), and professional competence (mean score 2.34). Emotional support, on the other hand, received the lowest score (0.96), according to the patients.

Similarly, providers also felt that the extent of PCC implementation varied across PCC dimensions. The providers, as can be seen in Fig. 1, gave the highest scores to these dimensions: respect for patient’s values and needs, communication skills, and emotional support (2.47. 2.48 and 2.54 respectively). Continuity and transition in treatment, accessibility of providers and physical comfort received the lowest score (2.06, 2.13 and 2.19 respectively), according to the providers.

FT patients and providers differed in their perceptions of provision of the various dimensions of PCC and of which of the dimensions were more or less applied, in regard to three out of 10 dimensions (Fig. 1). Overall, FT patient scores tended to be lower than those of the providers, except for continuity of treatment and professional competence, in which the scores of the providers were slightly lower. The largest gap between FT patients’ perceptions and the providers’ perceptions was in emotional support (1.58), followed, but unmatched with, by respect for patient’s values and needs and provision of information and explanations (a gap of 0.45–0.55).

The IVF units varied in the patient overall scores (mean scores ranged between 1.89 and 2.49) and in the scores for the various dimensions of PCC (Table 4). However, the lowest score in each and every IVF unit was the score the patients gave to emotional support (0.65–2.18). The range between the highest and the lowest scores per unit in emotional support exceeded 1.5 while in almost all the other dimensions it did not exceed 0.88.

**Table 4:** Scores^a^ on a 0–3 scale provided by FT patients on their perception of the various dimensions of PCC, by IVF units

PCC Dimensions	IVF units								
	1	2	3	4	5	6	7	8	SD^b^
Accessibility of providers	1.46	1.99	1.88	2.27	1.96	2.69	2.62	1.99	0.5–0.9
Information and explanation	1.81	1.85	2.25	1.87	1.94	2.47	2.25	1.87	0.4–0.6
Communication skills of providers	2.17	2.30	2.42	2.27	2.45	2.74	2.26	2.28	0.4–0.6
Involvement of patient in treatment	1.98	2.13	2.24	2.05	2.14	2.52	2.16	2.05	0.6–0.8
Respect for values and needs	1.86	2.01	2.28	2.08	2.33	2.55	2.13	1.77	0.5–0.8
Continuity and transition in treatment	2.11	2.04	1.90	2.00	1.89	2.36	2.23	2.34	0.4–0.6
Professional competence	2.16	2.20	2.22	2.27	2.20	2.51	2.15	2.39	0.3–0.5
Care organization	1.76	1.95	2.06	2.55	2.01	2.64	2.54	2.40	0.4–0.8
Physical comfort	1.92	1.59	2.04	2.04	1.79	2.20	1.79	2.31	0.6–0.8
Emotional support	1.21	1.27	1.72	0.85	1.02	2.18	1.78	0.65	0.8–1.1
Total	1.89	1.97	2.14	2.00	2.02	2.49	2.18	2.00	0.3–0.5

^a^All scores represent significant differences at the level of *p* < 0.001

^b^The range of standard deviation values of the average scores in all IVF units in each dimension

Furthermore, the IVF units varied in the gaps between patients and providers perceptions of the 10 dimensions of PCC (Table 5). While the gaps between the scores provided by FT patients and providers in the various dimensions varied between 0 (i.e, no gap) to 0.78, the gaps per unit between the patients and the providers in the emotional support dimension was larger and varied between 0.82 and 1.70.

**Table 5:** Gaps^a^ between the scores on a 0–3 scale provided by FT patients and providers on their perception of the various dimensions of PCC, by IVF units

PCC Dimensions	IVF units^b^							
	1	2	3	4	5	6	7	8
Accessibility of providers	0.60	0.08	0.67	0.30	0.31	0.25	0.04	0.51
Information and explanation	0.16	0.56	0	0.78	0.50	0.15	0.45	0.39
Communication skills of providers	0.16	0.15	0.21	0.27	0.09	0.03	0.16	0
Involvement of patient in treatment	0.23	0.20	0.44	0.47	0.19	0.01	0.11	0.35
Respect for values and needs	0.14	0.34	0.25	0.40	0.14	0.30	0.17	0.72
Continuity and transition in treatment	0.28	0.31	0.06	0.15	0.05	0.25	0.35	0.16
Professional competence	0.35	0.15	0.38	0.01	0.17	0.10	0.23	0.21
Care organization	0.24	0.13	0.29	0.08	0.04	0.13	0.01	0.12
Physical comfort	0.11	0.26	0.33	0.10	0.24	0.25	0.47	0.14
Emotional support	1.19	1.23	0.85	1.40	1.00	0.82	1.20	1.70
Total	0.02	0.23	0.08	0.42	0.26	0.11	0.34	0.39

^a^All gap scores represent significant differences at the level of *p* < 0.001

^b^The result of the subtraction of the patients’ scores from the provider scores in absolute values

## Discussion

Four primary findings emerge from this study. First, FT patients felt that not all dimensions of PCC were implemented equally. While it seemed that the FT patients in the current study had high assessments of r the professional abilities of the providers treating them (which included their communication skills and professional competence), and the organization of the treatment, they had low assessments of the providers’ performance in providing them with the emotional support they felt they needed.

Although one might hypothesize that patients’ experience in regard to PCC in FT would differ between countries, it seems that our findings regarding Israeli FT patients are consistent with those reported regarding FT patients in other countries [24, 30]. In a recent international comparison of PCC dimensions scores, which included New Zealand, the Netherlands, Slovakia, Portugal, Iran and Slovenia, communication skills and professional competence were scored the highest, while in most countries continuity and transition in treatment received the lowest score [31]. This might be partially explained by the high appreciation patients, specifically in Israel but in other countries as well, have of their providers. This may be especially true in FT.

The second main finding was that providers underestimate FT patients’ need for certain dimensions of PCC, as can be reflected in the gaps between the FT patients and providers scores in these three PCC dimensions: information and explanations, respect for values and needs and emotional support. Most importantly, these gaps indicate a disagreement between FT patients and providers regarding the degree to which these dimensions were implemented. Clearly, FT patients’ perception of the care they received, on the one hand, and the providers’ perception of the care that they provide, on the other, do not always conjoin in fundamental dimensions of PCC.

The third principal finding was that there were substantial differences among IVF units in their scores on the various dimensions of PCC. The healthcare system in Israel, including FT, is required to adhere to all standards regarding patient experience and PCC, including, for example, addressing patients’ emotional needs, and patients’ involvement in care [32]. Given that, the findings regarding the differences across units between FT patients and provider scores, which were more prominent in the provision of emotional support, are surprising. We have no ready explanation for the FT patient-provider differences in the perception of emotional support between IVF units. This finding was not expected and therefore requires confirmation. It is worth mentioning that the Ministry of Health in Israel is currently monitoring IVF units, and examines several components related to PCC and emotional support, such as the standard staffing of social workers and psychologists in the units, providing information and explanations, and signing an informed consent form before treatment. Linking the abovementioned data with findings of PCC surveys, such as the current study, might shed light on some of the intervening factors related to patient experience and PCC in FT.

The last main finding was the prominent patient-provider gap concerning emotional support. Not only did FT patients give emotional support the lowest score among the various PCC dimension, while providers scored it the highest, but of all dimensions, emotional support captured the largest gap between patients and providers in all IVF units. One possible explanation might be that providers assume they manage to fulfill their patients’ need for certain dimensions of PCC, while their patients do not feel so. Another possible explanation might be that providers do not necessarily think it is part of their duty to address patients’ emotional needs. In a study examining attitudes of physicians in fertility clinics in 15 countries regarding the emotional needs of their patients, less than half (45%) thought that being able to address the emotional needs of patients was necessary, and 72% reported needing improvement in their ability to identify the needs of patients for emotional support [33]. Moreover, in a recent cross-sectional exploratory study of fertility physicians, the authors found that although the majority of providers believed emotional conditions negatively impact pregnancy success, most of them did not screen patients for depression or anxiety [34].

In FT and other medical fields as well, it seems that providers fail in recognizing the emotional needs of their patients. In a study on cancer patients’ emotional distress, the oncologists recognized the presence of severe distress in only about a third of the severely distressed patients, and the oncologists’ recommendations for supportive counselling did not correlate with patient distress [35]. Indeed, The European Society of Human Reproduction and Embryology (ESHRE) published a guideline for routine evidence-based emotional support in fertility treatment for all clinic staff (physicians, nurses, midwifes, counsellors, social workers, psychologists, embryologists and administrative personnel), which include 120 recommendations related to 12 key question. The guidelines specify how to enable couples, their families and their providers to optimize FT and manage the psychological and social implications of infertility and its treatment on optimal management of routine emotional care by all provider. For example the guidelines highlight the importance of being aware that patients′ emotional stress fluctuates during a treatment cycle, with peaks at the oocyte retrieval, the embryo transfer and the waiting period before the pregnancy test [36]. Having said that, it is important to note it was not in the scope of the current study to attempt to understand the reasons for the emotional distress of women in FT, for which the providers themselves may not be solely responsible, but to focus on PCC due to its beneficial association with patient wellbeing.

The observed differences and gaps between what FT patients feel about their care and what providers assume they provide, particularly the gap in emotional support, requires special attention and identifies the need for specific feedback for providers regarding how their professional performance meets the needs of FT patients. Attempts at improving PCC begin by giving appropriate feedback to providers [37] and gaining an insight into the discrepancies between patient and providers perceptions [6]. Using PCC feedback tool, which is based on the patient perspective, is critical to identifying areas of care where improvements are needed, since patients are placed in the best position to decide whether care is consistent with their values, preferences and needs. They also know best whether they received the level of information they desire, and whether they understood the information and can recall it [38].

Accordingly, in the current study each unit participating was presented with a personal feedback report, containing the scores of the patient in that specific unit on the 10 dimensions of PCC, and a comparison to the other units. Thus, providers and unit managers could examine the scores on each dimension and each element, and see the focal points required for change, and what should be preserved in their practice, in order to improve the PCC of the patient being treated in their unit.

In line with the above, the four elements comprising the emotional support dimension, that should be addressed are: being informed about the psychological impact of treatment; giving the opportunity to consult a counselor who is familiar with problems connected with treatment; receiving information on support group for FT patients; and ensuring that a partner and/or family members are given an information brochure. It is important to examine which effective interventions can be developed in order to improve the PCC in fertility treatment, since even extensive interventions involving providers in fertility clinics [39], or tailored expectant management involving patient and providers in fertility clinics [40] don’t always have the desired impact of improving PCC. In a review describing the optimal IVF treatment in 2020, the authors argue that patient emotional vulnerability can be tackled by screening for emotional distress before treatment unset, referrals for emotional support and elimination of barriers to acceptance of such support, and implementing a routine care flowchart that identifies the specific stages of treatment when emotional support should be provided [41].

## Limitations

This study has several limitations. First, the data collected in the current study are limited to women treated in one of the eight sampled units, out of 25 IVF units currently operating in Israel. As mentioned above, a random sampling method from patient lists was not possible, for reasons of reluctance on the part of the managers of the participating IVF units to reveal the list of patients receiving treatment in their unit. Therefore, a two-stage sampling method was used: unit selection and quota sampling in each unit. For these reasons, it is possible that the women who participated in the study did not represent all women undergoing FT in Israel.

However, the units were sampled so that they reflected the diversity in their characteristics (location, magnitude and ownership), and it can be presumed, with due caution, that the women participated in the current study represent the variety of the characteristics of the women undergoing FT in Israel, for example in terms of their religion, their country of birth and their place of residence. In order to delve into specific socio-cultural differences in PCC perceptions, further research is needed, with a greater representation of women from minority groups in Israel.

Furthermore, the current study design, which included data collection from each patient at one time point, did not allow causality relationships to be examined. Longitudinal research could lead to better understanding of cause and effect relationships in the context of PCC of women undergoing FT. However, the findings from the current study are valuable and might serve as fundament for future research. Subsequent studies will benefit from approaching all units operating in Israel, in terms of deepening the understanding of the differences between IVF units.

## Conclusions

Providers appear to underestimate FT patients’ needs for information, respect, and emotional support. The observed differences between what patients feel about the care and what providers assume they provide, especially regarding emotional support, indicates a need for ongoing, specific feedback to providers regarding the patient-centeredness of the care they provide. The particularly large patient-provider gap regarding the provision of emotional support highlights the importance of increasing the attention given to the psychological impact of fertility treatment and of giving patients an opportunity to consult a counselor who is familiar with problems associated with fertility treatments.

## Policy recommendations

Fertility problems significantly reduce the quality of life and increase anxiety and depression levels. Patient-centeredness is associated with patient wellbeing, quality of life and reduced distress. In order to help patients get through fertility treatments with reasonable wellbeing, we see it as the duty of health care providers, to improve the patient-centeredness of the care that they provide. Our recommendations hereafter concentrate on the “what, how and who” that should be considered in order to do so.

It is essential, but not sufficient, to increase attention to the importance of the patient-centeredness and the patient-provider gap regarding this issue, and the current study has already increased attention to this. It is also important to assist providers and unit managers in receiving ongoing feedback regarding the extent to which their professional performance meets the needs of their patients.

The current study’s scope covered only eight participating units out of the 25 IVF units operating in Israel, and data were collected at only one point in time. We recommend disseminating the current research tool nationally, to all IVF units operating in Israel and guiding the providers and unit managers about conducting an ongoing patient survey. This could be done through a collaboration of the Ministry of Health’s program for monitoring the IVF units, and the corresponding authors. Sharing the individual units’ scores, preferably by the providers themselves, with the general public, might help patients in the process of making an informed decision about the unit where they will receive treatment. This might be done via the hospitals’ internet site, or other online platforms.

In order to improve the patient-centeredness we recommended appointing a professional in each unit who, as part of his or her job in the unit, will be responsible for promoting improvements in patient-centered care in the unit. This appointee should see it as his or her mission to bring the patient-centeredness perspective to bear on all processes and procedures being done in the units, e.g. distributing guidance pamphlets to patients and allocating designated spaces in the unit in case of renovations. It is important for the unit manager to support this activity and that professional guidelines will be developed by existing hospital resources units, such as the hospital social service unit. This appointee might also be responsible for the ongoing feedback process and for presenting the findings at the national level, especially in relevant professional conferences, such as the Annual Conference of the Israel Fertility Association (IFA). It might be beneficial to place in this position a professional who is part of the clinical team, in order to enable the other providers to identify with and internalize the importance of patient-centeredness.

It is also very important that a mental health professional (psychologist or social worker) be assigned to each and every IVF unit, as part of the unit’s permanent staff. Presently, the majority of units in Israel do not have a mental health professional assigned to them on a permanent, full-time basis. As a result, they usually involve a mental health professional only in urgent times, when acute aid is needed. This structural feature of the units makes it very difficult to give the patients the emotional support they need.

The required mental health professional position should be responsible for initial intake for each new patient, and for giving emotional treatment within the unit. His or her presence in the unit should be prominent and significant, including participation in meetings or professional discussions in the unit, such as daily professional staff meetings regarding treatment outcomes. The mental health professional can also support the patient-centeredness’ appointee, and initiate and organize professional seminars on relevant issues. It is important to note that a mental health professional who is an integral part of the FT team, can also support the providers themselves, giving them the support they need to continue caring for their patients and reducing burnout.

## Data Availability

The datasets used and/or analyzed during the current study are available from the corresponding author on reasonable request.
